# Unexpectedly high piezoelectricity of Sm-doped lead zirconate titanate in the Curie point region

**DOI:** 10.1038/s41598-018-22566-5

**Published:** 2018-03-07

**Authors:** Shruti B. Seshadri, Michelle M. Nolan, Goknur Tutuncu, Jennifer S. Forrester, Eva Sapper, Giovanni Esteves, Torsten Granzow, Pam A. Thomas, Juan C. Nino, Tadej Rojac, Jacob L. Jones

**Affiliations:** 1 Department of Materials Science and Engineering, University of Florida, Gainesville, Florida 32611 USA; 2 Department of Materials Science and Engineering, North Carolina State University, Raleigh, North Carolina 27695 USA; 3 Department of Chemistry, University of Florida, Gainesville, Florida 32611 USA; 4 School of Chemical and Process Engineering, University of Leeds, Leeds, LS2 9JT United Kingdom; 5 Institute of Materials Science, Technische Universität Darmstadt, 64287 Darmstadt, Germany; 6 Materials Research & Technology Department, Luxembourg Institute of Science & Technology, L-4362 Esch, Alzette Luxembourg; 7 Department of Physics, University of Warwick, Coventry, CV4 7AL United Kingdom; 8 Jozef Stefan Institute, Jamova cesta 39, 1000 Ljubljana, Slovenia; 9 Present Address: Intel Corporation, Oregon, USA

## Abstract

Large piezoelectric coefficients in polycrystalline lead zirconate titanate (PZT) are traditionally achieved through compositional design using a combination of chemical substitution with a donor dopant and adjustment of the zirconium to titanium compositional ratio to meet the morphotropic phase boundary (MPB). In this work, a different route to large piezoelectricity is demonstrated. Results reveal unexpectedly high piezoelectric coefficients at elevated temperatures and compositions far from the MPB. At temperatures near the Curie point, doping with 2 at% Sm results in exceptionally large piezoelectric coefficients of up to 915 pm/V. This value is approximately twice those of other donor dopants (e.g., 477 pm/V for Nb and 435 pm/V for La). Structural changes during the phase transitions of Sm-doped PZT show a pseudo-cubic phase forming ≈50 °C below the Curie temperature. Possible origins of these effects are discussed and the high piezoelectricity is posited to be due to extrinsic effects. The enhancement of the mechanism at elevated temperatures is attributed to the coexistence of tetragonal and pseudo-cubic phases, which enables strain accommodation during electromechanical deformation and interphase boundary motion. This work provides insight into possible routes for designing high performance piezoelectrics which are alternatives to traditional methods relying on MPB compositions.

## Introduction

Piezoelectric materials exhibit electromechanical coupling and thus generate a voltage in response to a mechanical strain and vice versa. This coupling makes piezoelectrics extensively used as electroactive materials in the transducer industry^1^. Due to its high electromechanical sensitivity and piezoelectric coefficients, the solid solution of lead zirconate titanate (PZT), PbZr_(1−x)_Ti_x_O_3_^2,3^, has been the most commonly used commercial piezoelectric material for the last 60 years^1,4^.

Understanding the origin of the high piezoelectric response in PZT is a topic of fundamental importance and has been the subject of much investigation. However, new mechanisms and effects are still being discovered due to the plethora of available chemical compositions, dopants, temperature regimes, polymorphic phases and phase transitions^5^. It is generally accepted that high piezoelectric properties are obtained in PZT by using a zirconium to titanium ratio (Zr:Ti) close to the morphotropic phase boundary (MPB) at Zr:Ti 52:48^6,7^ and by the use of donor dopants^6,8,9^. Donor dopants are aliovalent substituents whose valence is greater than the valence of the host cation. Temperature also plays a role determining the magnitude of the piezoelectric response, because thermal energy influences domain wall motion and can induce phase transitions^6^.

The present work reports the unexpected discovery of high piezoelectric coefficients in PZT when chemically modified with Sm as a substituent for Pb. The high piezoelectricity is observed at elevated temperatures in the region of the Curie temperature (*T*_c_) At these temperatures, the piezoelectric coefficients of Sm-modified PZT reach 915 pm/V, approximately twice the values measured using the common dopants (477 pm/V for Nb and 435 pm/V for La). It is shown that, unlike the other dopants studied, Sm alters the ferroelectric (tetragonal) to paraelectric (cubic) phase transition characteristics in PZT. This mechanism for enhancing piezoelectricity in the classic PZT system has not been discovered previously because high-temperature piezoelectric coefficient measurements have been rare until recent years^10^. Additionally, the current generation high resolution synchrotron XRD capabilities^11^ enable the determination of the phase assemblage at high temperatures.

## Results

Compositions studied are PZT 50:50, PZT 45:55 and PZT 40:60 (where numbers denote mol% of Zr and Ti, respectively) doped with 2 atomic percent of Sm, La or Nb. The dopants La and Nb were chosen to provide a baseline for comparing Sm doping as they are the most commonly used donor dopants in commercial PZT. The nominal chemical formulae of the doped materials are: (i) Sm: Pb_0.97_Sm_0.02_Zr_0.5_Ti_0.5_O_3_, Pb_0.97_Sm_0.02_Zr_0.45_Ti_0.55_O_3_, Pb_0.97_Sm_0.02_Zr_0.4_Ti_0.6_O_3_, (ii) La: Pb_0.97_La_0.02_Zr_0.5_Ti_0.5_O_3_, Pb_0.97_La_0.02_Zr_0.45_Ti_0.55_O_3_, Pb_0.97_La_0.02_Zr_0.4_Ti_0.6_O_3_ and (iii) Nb: PbZr_0.4875_Ti_0.4875_Nb_0.02_O_3_, PbZr_0.4388_Ti_0.5363_Nb_0.02_O_3_ and PbZr_0.39_Ti_0.585_Nb_0.02_O_3_.

**Figure 1:**
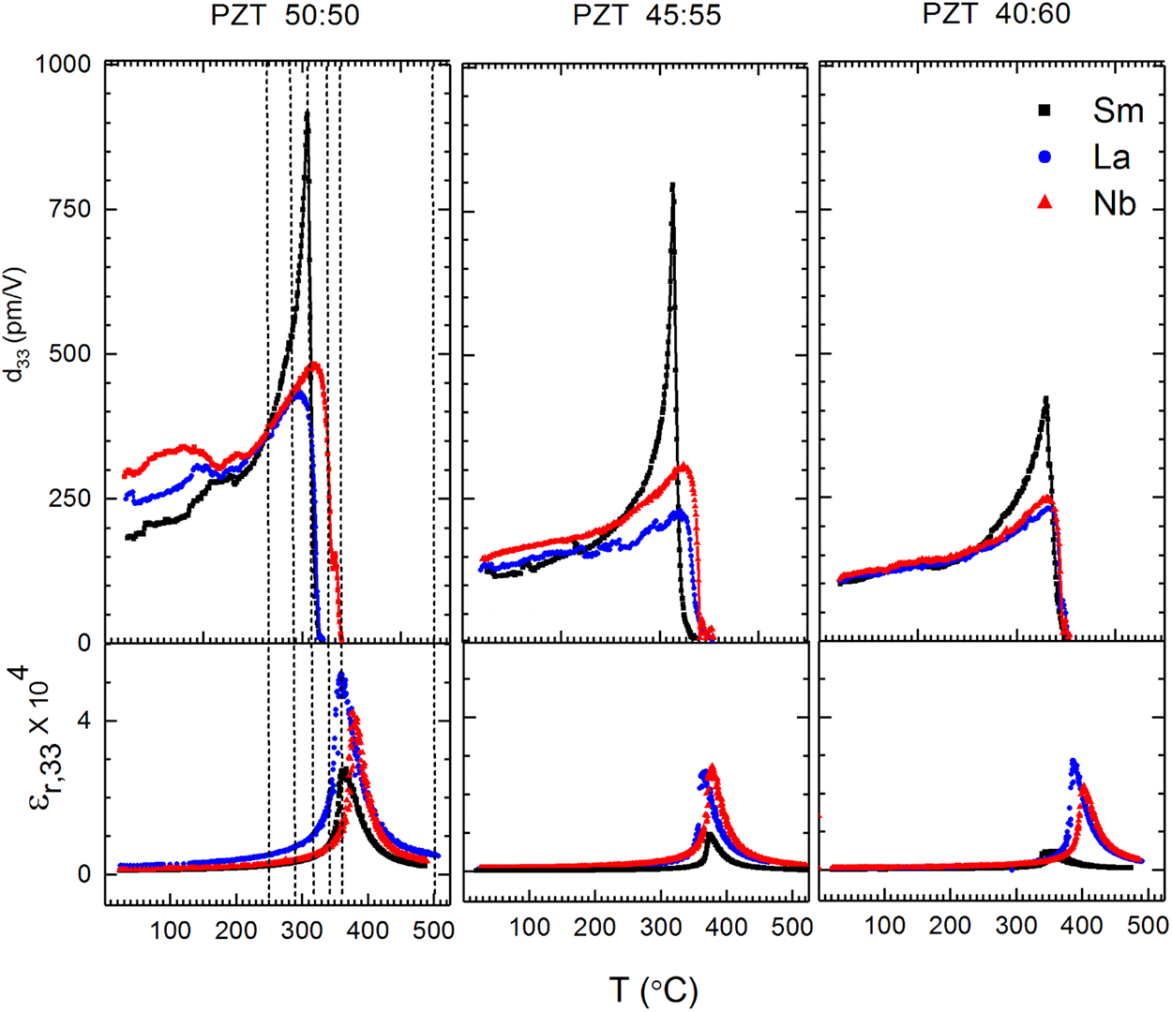
Variation of *d*_33_ and *ε*_*r*,33_ as a function of temperature for various PZT compositions. The lines in the left panel show critical temperatures selected for further structural analysis.

The values of the converse longitudinal piezoelectric coefficient, *d*_33_ and permittivity, *ε*_*r*,33_ in all compositions as a function of temperature are shown in Fig. 1. For all Zr:Ti ratios, the *d*_33_ values of the Sm-, La- and Nb-doped PZT samples increase with increasing temperature until ≈250 °C. Above this temperature, the behavior of the Sm-doped compositions diverges from that of the La- and Nb-doped compositions. In the La- and Nb-doped compositions, the increase in *d*_33_ with temperature continues after ≈250 °C, until the maximum *d*_33_ is reached. In the case of Sm-PZT, a dramatic and unexpected increase in *d*_33_ is observed as a function of temperature. For each Zr:Ti ratio, the maximum *d*_33_ observed in Sm-doped PZT is approximately twice that observed in either the La- or the Nb-doped materials.

**Figure 2:**
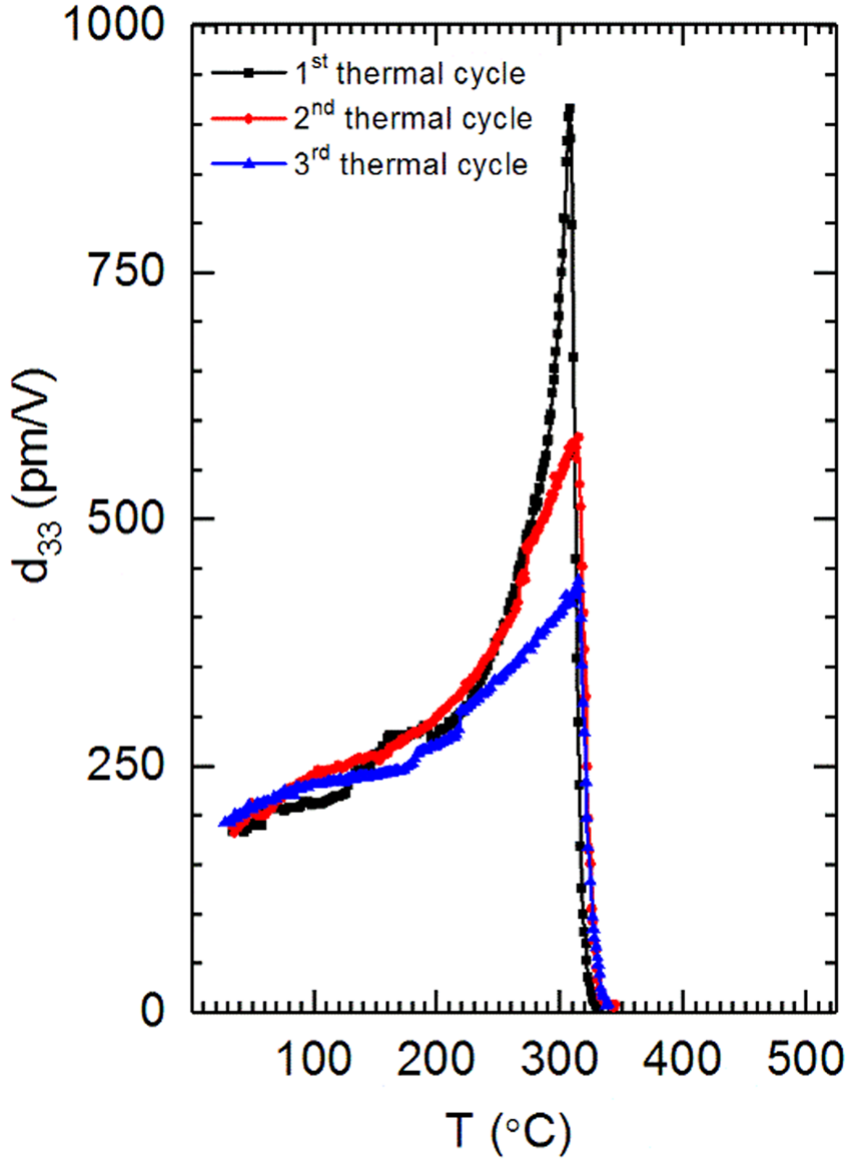
Behavior of *d*_33_ as a function of temperature during the first, second and third thermal cycles for 2 at% Sm-doped PZT 50:50.

The effects of thermal history on this behavior are also explored by (re)poling and measuring properties after successive thermal cycles. Heating the PZT compositions from room temperature to the depoling temperature is referred to here as a thermal cycle. After thermal cycling, Sm- doped PZT 50:50 was again poled to saturation at room temperature and its *d*_33_ behavior as a function of temperature determined for a second time. As shown in Fig. 2, in contrast to the maximum *d*_33_ obtained after the first cycle (915 pm/V), the maximum *d*_33_ observed during the second thermal cycle is 583 pm/V. This is a decrease of 332 pm/V from the first thermal cycle. However, it is still higher than the maximum *d*_33_ observed in La-doped PZT 50:50 (435 pm/V) and Nb-doped PZT 50:50 (475 pm/V) during their first thermal cycles (see Fig. 1). After the completion of the second thermal cycle, Sm-doped PZT 50:50 was poled to saturation for a third time and the *d*_33_ behavior as a function of temperature was again measured. The maximum *d*_33_ observed during the third thermal cycle is 438 pm/V (Fig. 2). This value is 477 pm/V lower than that observed during the first thermal cycle of Sm-doped PZT and is thus similar to the first-cycle behavior of the other dopants studied (see Fig. 1). This thermal cycling behavior was found to be repeatable across several samples of the same composition. It is important to note that the sample was not annealed to processing temperatures between thermal cycles and, thus, it is not expected that the sample would regain full properties. The reduction in the peak of the piezoelectric coefficient with thermal cycling could be due to irreversible electric-field-dependent or temperature-dependent changes in the sample such as changes in phase fractions, domain wall density, domain wall mobility, or concentration or distribution of point defects. This cycle-dependent behavior suggests an extrinsic effect is the origin of the high properties, since the magnitude of the effect can be adjusted by cycling.

To obtain insight into the structural changes taking place in the materials as a function of temperature, X-ray diffraction (XRD) patterns were measured at a high resolution beamline at a synchrotron source. Diffraction patterns of Sm-, La- and Nb-doped PZT 50:50 were measured at 250 °C, 290 °C, 308 °C, 332 °C, 360 °C and 500 °C. These temperatures were selected to analyze the structural behavior around the maxima in *d*_33_ and *ε*_*r*,33_ as a function of temperature (see Fig. 1, dashed lines).

**Figure 3:**
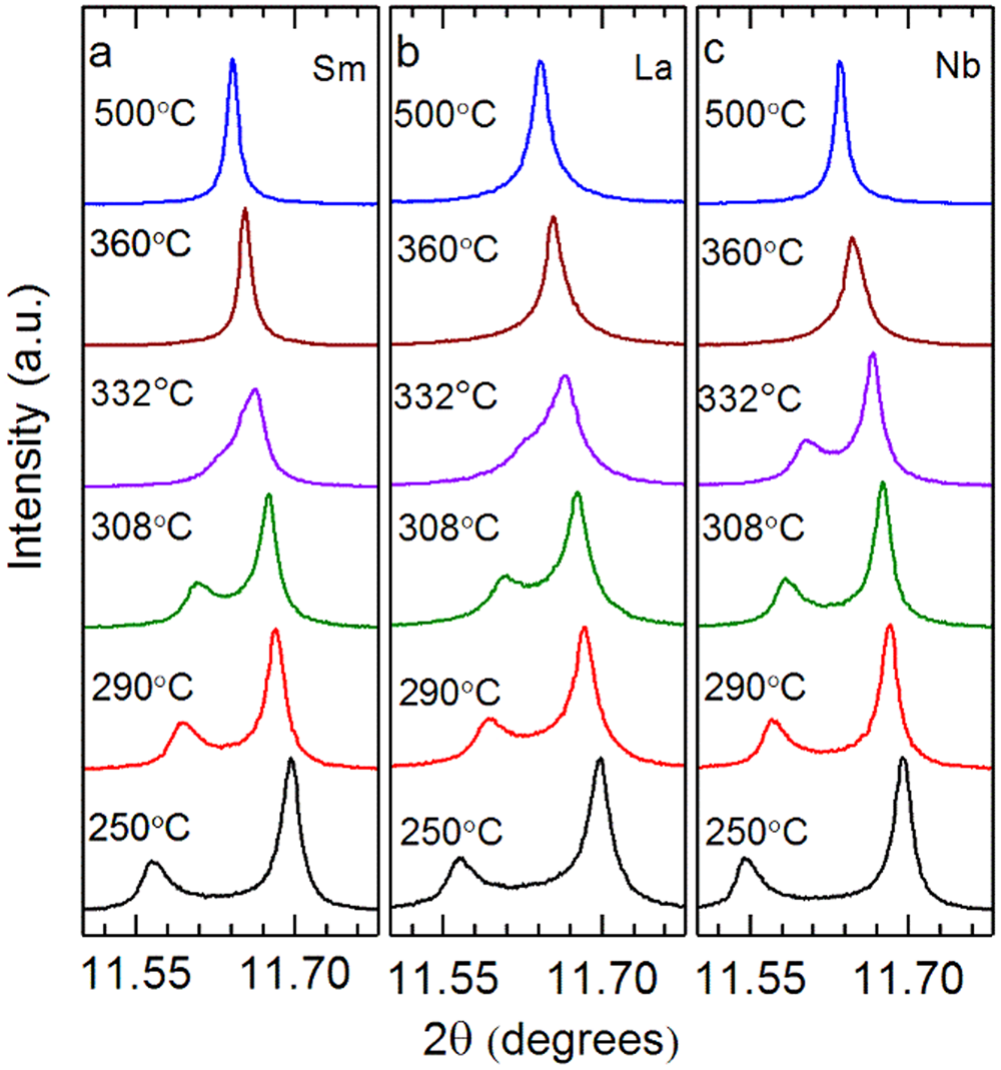
Selected regions of high resolution synchrotron XRD patterns showing the {002} diffraction profile for (**a**) Sm-, (**b**) La- and (**c**) Nb-doped PZT 50:50.

Selected regions of the XRD patterns containing the 002 and 200 reflections of the materials is shown in Fig. 3. With increasing temperature, the 002 and 200 reflections of the ferroelectric tetragonal phase shift closer together and finally merge into a single 002 reflection above *T*_c_^6^. At 308 °C and 332 °C, some extra intensity is observed between the 002 and 200 reflections in the Sm-doped composition. It is proposed that a single tetragonal phase insufficiently describes the diffraction pattern of Sm-doped PZT 50:50 at 308 °C and 332 °C due to the presence of an additional polymorph.

To examine this more closely, the diffraction profile near the 200-type reflections acquired at 332 °C was modeled using the peak-fitting procedure described in the Methods section for the three compositions. The 332 °C patterns were selected because the observed extra intensity in the Sm-doped PZT pattern is the most pronounced at this temperature. For each pattern, peaks were modeled using either two peaks or three peaks. The two-peak model represents the tetragonal 002 and 200 reflections. For the three-peak model, the additional (third) peak was located between the tetragonal 002 and 200 reflections and intended to test the possibility of a third reflection from a secondary polymorph, e.g. the 200 reflection from a closely cubic (pseudo-cubic) phase.

**Figure 4:**
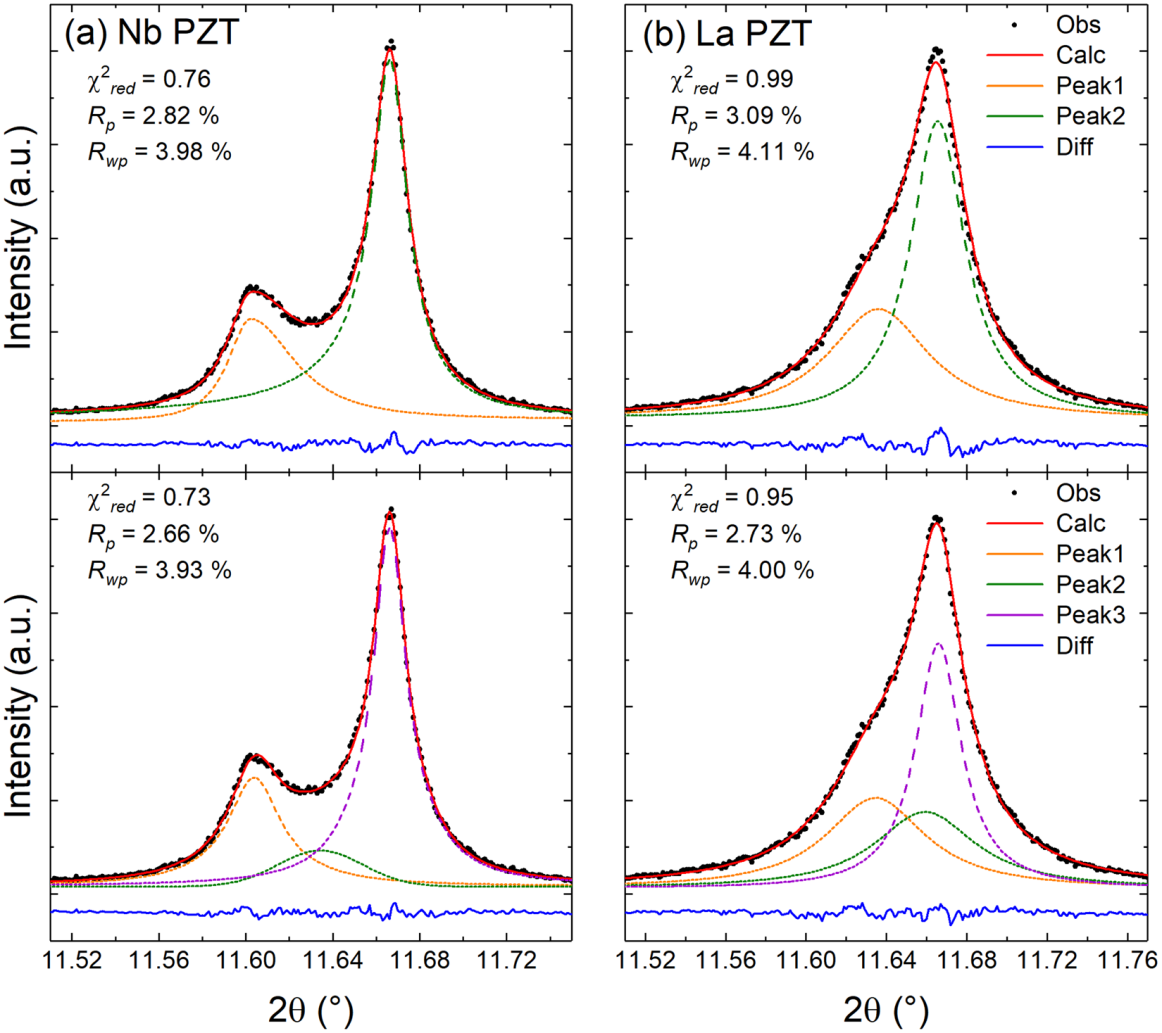
Profile fits of the {200} diffraction profiles at 332 °C of (**a**) Nb and (**b**) La -doped PZT. For the Nb-doped PZT, 2 asymmetric PVII peaks were used. 2 Lorentzian peaks were used for the La -doped PZT. The difference patterns are shown in blue below the profiles.

There are several observations that can be made from these profile fits. The profiles for Nb- and La- doped PZT (Fig. 4) are characteristic of a ferroelectric tetragonal structure with twin-related domain wall scattering between the 002 and 200 reflections^12^. In the case of the La-doped PZT, two symmetric functions are sufficient to model the data, however, an asymmetric Pearson VII (PVII) function can also be used to approximate diffuse scattering (Supplementary Information Fig. S1). Regardless of which function type is used (symmetric or asymmetric), the patterns for the Nb- and La- doped PZT are adequately described using a two-peak model. The addition of a third peak increases the goodness-of-fit only slightly which, in combination with the observed profile, is insufficient justification for the presence of three peaks.

**Figure 5:**
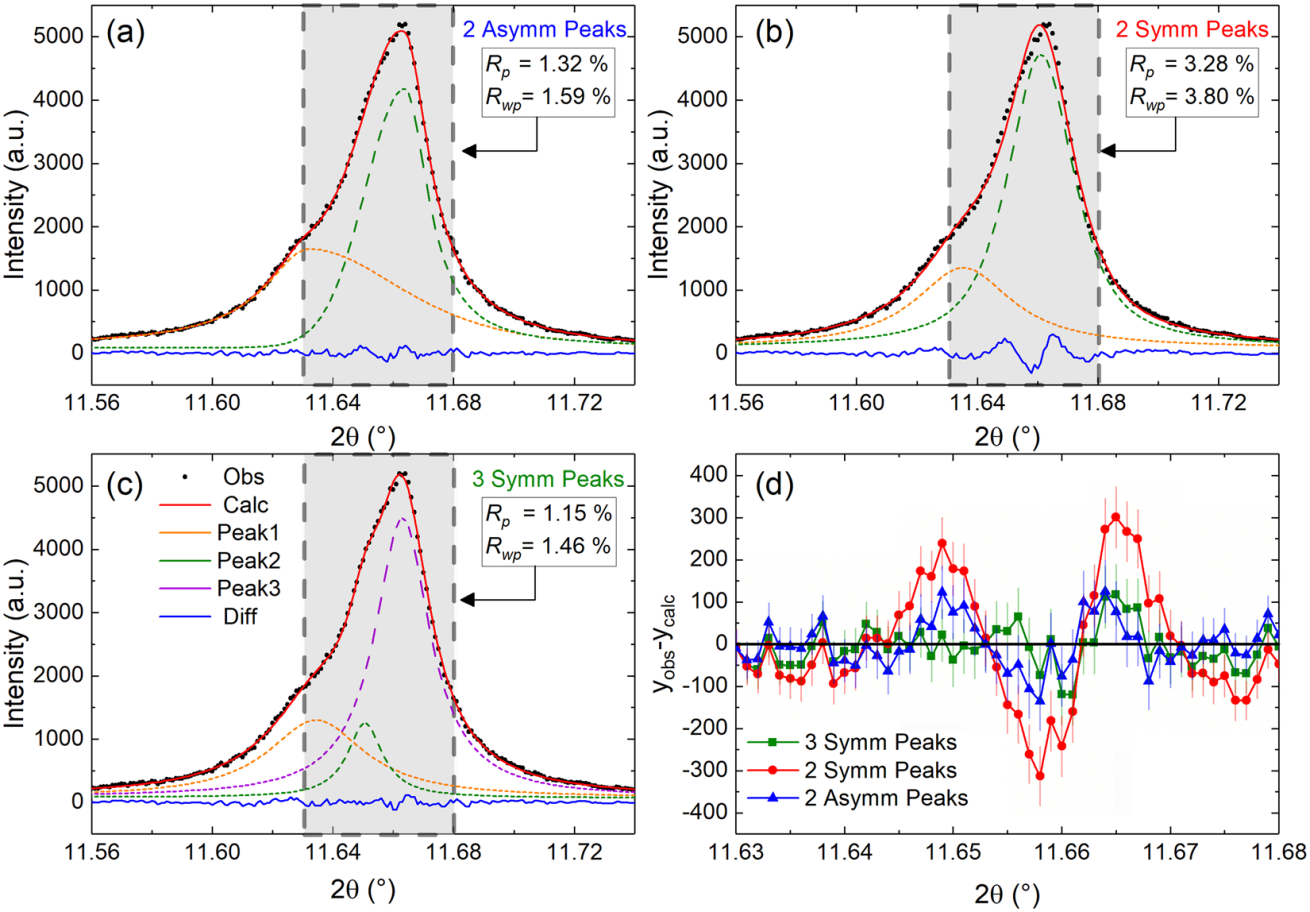
Multiple profile fits for the Sm-doped PZT 50:50 at 332 °C. The best fit is obtained by using three symmetric peaks to represent a phase coexistence of tetragonal and pseudo-cubic. The shaded gray region highlights where a distinct shoulder is present on the left-hand side of the tetragonal 200 reflection. The *R*_p_ and *R*_wp_ values are calculated from the 2θ range within the gray shaded box.

The Sm-doped pattern shown in Fig. 5 expresses a distinct shoulder on the left-hand side of the tetragonal 200 reflection. Applying two asymmetric functions to fit the Sm-doped PZT data (Fig. 5a) leads to a better fit than using two symmetrical functions (Fig. 5b). However, the use of asymmetric peaks, as done in ref.^12^ and for the Nb-doped sample, cannot model the distinct shoulder of the left-hand side of the 200 tetragonal reflection. Additionally, using two asymmetric peaks leads to elongated tails which are unphysical. Therefore, the observation of a third peak in the present work is indicative of a third reflection, i.e., not domain wall scattering. This is because diffuse scattering from domain walls does not lead to a distinct peak, rather taking on an entirely different functional form^12^. The superior fit of the diffraction pattern to the three-peak model is demonstrated in Fig. 5c and reinforced by the *R*_p_ and *R*_wp_ values as well as the difference plots in Fig. 5d. For completeness, Supplementary Information Fig. S1 applies the methodology used in ref.^12^ and demonstrates that the residual intensity between the Bragg peaks reflects a third Bragg peak rather than domain wall scattering. Qualitatively, it can be seen in Fig. 5 that the inclusion of the third peak allows an overall fit to the pattern that captures the shoulder on the left-hand side of the tetragonal 200 reflection. The goodness-of-fit values further show that the three peak model yields a superior fit. Therefore, only two peaks are needed to sufficiently describe the {200} diffraction profile of the Nb- and La-doped patterns, whereas three are necessary to describe the profile of the Sm-doped sample. Supplementary Information Figs S1 and S2 also show that asymmetric profiles cannot adequately capture the qualitative features of this shoulder in Sm doped PZT, reinforcing its identification as an extra reflection.

The various polymorphs which may give rise to the additional reflection in Sm-doped PZT are now considered. In addition to the tetragonal phase, the other phases that have been previously observed in the PZT phase diagram are rhombohedral, monoclinic and cubic^7^. From the phase diagram, it can be seen that for PZT 50:50 the rhombohedral phase is only present at low temperatures. This makes it unlikely that the second polymorph detected at 332 °C is rhombohedral. It is also unlikely that the second polymorph is monoclinic because the symmetry of ferroelastic crystal structures tends to increase with temperature. Thus, after eliminating rhombohedral and monoclinic phases, the extra intensity at 332 °C between the 002 and 200 reflections is most likely due to the presence of a secondary polymorph that is either cubic or pseudo-cubic. Because of the small distortions and resulting peak overlap, the precise lattice type of the secondary polymorph cannot be definitively determined and herein is referred to as a pseudo-cubic phase.

**Figure 6:**
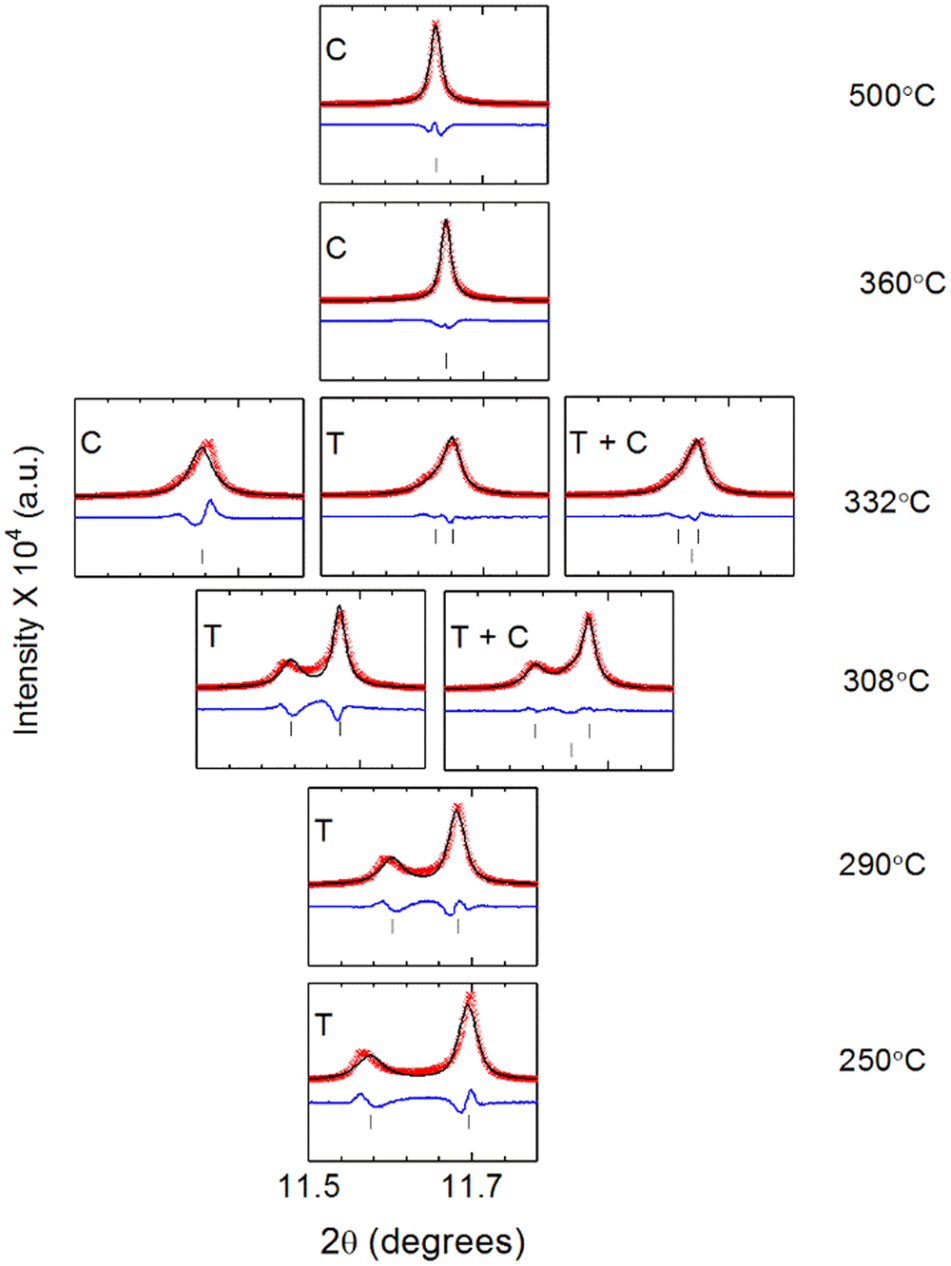
Rietveld refinements of 2 at % Sm-doped PZT 50:50 fit to a cubic model (332 °C, 360 °C, 500 °C), tetragonal model (250 °C, 290 °C, 308 °C, 332 °C) and a mixed phase model consisting of the tetragonal *P*4*mm* and (pseudo−) cubic *Pm*$\bar{3}\,$*m* phases (308 °C, 332 °C).

**Figure 7:**
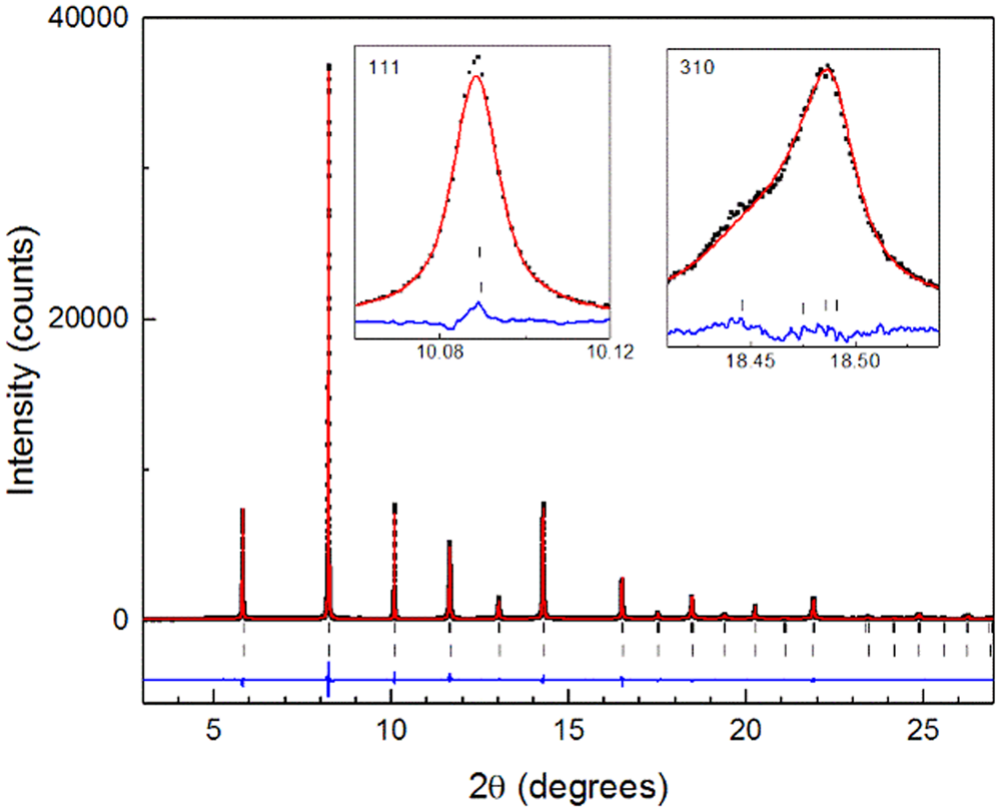
Full Rietveld refinement of Pb_0.97_Sm_0.02_Ti_0.5_Zr_0.5_O_3_ fitted to a mixed phase model of tetragonal *P*4*mm* and (pseudo−) cubic *Pm*$\bar{3}\,$*m* phases at 332 °C. Measured diffraction pattern is represented by black markers. The red and blue lines are the calculated model and difference pattern, respectively. Insets show the single 111 reflection and a representative higher order reflection.

In order to quantify the phase fraction of the pseudo-cubic phase present in Sm PZT 50:50 (Pb_0.97_Sm_0.02_Ti_0.5_Zr_0.5_O_3_) at all temperatures, crystallographic refinement was undertaken using the Rietveld method. The refinement of Pb_0.97_Sm_0.02_Ti_0.5_Zr_0.5_O_3_ to a mixed-phase model (containing tetragonal *P*4 *mm* and pseudo-cubic *Pm*$\bar{3}\,$*m* phases) is shown in Figs 6 and 7. A summary of the refinement results for the patterns measured at all temperatures is available in Table 1. Comprehensive crystal structure outputs of the refined data at all temperatures are given in Supplementary Tables S1–S3. Using a quantitative calculation^13^, it was found that the phase fraction of the pseudo-cubic phase in Pb_0.97_Sm_0.02_Ti_0.5_Zr_0.5_O_3_ at 332 °C is 31%. The origin of this secondary phase is discussed below.

**Table 1:** Summary of Rietveld refinement results of Pb_0.97_Sm_0.02_Zr_0.5_Ti_0.5_O_3_. The standard deviations for phase fraction are of the order of 0.1%.

Temperature (°C)	Space group	Phase fraction	a (Å)	c (Å)	Criteria of fit
250	*P*4*mm*	100%	4.054075(24)	4.095974(32)	*R*_p_ = 9.00%
290	*P*4*mm*	100%	4.058846(20)	4.086615(26)	*R*_p_ = 7.90%
308	*P*4*mm* *Pm* $\bar{3}\,$ *m*	71% 29%	4.060155(15) 4.067567(23)	4.083100(21)	*R*_p_ = 5.40%
332	*P*4*mm* *Pm* $\bar{3}\,$ *m*	69% 31%	4.065098(15) 4.067787(22)	4.073675(34)	*R*_p_ = 5.70%
360	*Pm* $\bar{3}\,$ *m*	100%	4.067774(12)		*R*_p_ = 8.64%
500	*Pm* $\bar{3}\,$ *m*	100%	4.072079(10)		*R*_p_ = 7.59%

Refinements at the other temperatures provide perspective as to how the different phases and phase fractions (for the coexisting phases) evolve with temperature. At 250 °C and 290 °C, the diffraction patterns were modeled using a single tetragonal phase. At these temperatures, it is difficult to ascertain whether there is any small phase fraction of the pseudo-cubic phase within the sample given the resolution of the instrument, because the area between the 002 and 200 reflections is dominated by domain wall scattering. It could be inferred that there is a small contribution from the pseudo-cubic phase at 290 °C, as this is the temperature where the sharp increase in *d*_33_ begins. The 002 and 200 reflections in the XRD patterns recorded at 308 °C and 332 °C certainly suggest the presence of the secondary phase. It is emphasized that several different models were originally employed to attempt fitting the diffraction patterns: a single phase pseudo-cubic model (C), a single phase tetragonal model (T) and a mixed-phase pseudo-cubic and tetragonal model (T + C). The refinement results suggest that the mixed-phase, pseudo-cubic and tetragonal model best describes the measured diffraction patterns acquired at 308 °C and 332 °C, consistent with the peak fitting results presented earlier (Figs 4 and 5). The diffraction patterns collected at 360 °C and 500 °C were modeled using a cubic phase since no peak splitting was observed in the 00*l* reflections.

To determine the character of the transition between ferroelectric tetragonal and paraelectric (pseudo-)cubic phase, the dielectric permittivity is analyzed in terms of Landau-Devonshire theory. Typically, the coexistence of tetragonal and pseudo-cubic phase would imply a first-order type transition. This is not observed here. From the Landau-Devonshire theory, the relationship between permittivity and temperature below and above *T*_c_ is given by Equations (1) and (2) respectively^14^.

1 $$\frac{1}{\in }=8{\rm{\beta }}({T}_{{\rm{C}}}-{\rm{T}})+\frac{3{{\rm{\gamma }}}^{\text{'}2}}{4{\rm{\delta }}}$$

2 $$\frac{1}{\in }={\rm{\beta }}({\rm{T}}-{T}_{{\rm{C}}})+\frac{3{{\rm{\gamma }}}^{\text{'}2}}{16{\rm{\delta }}}$$

**Figure 8:**
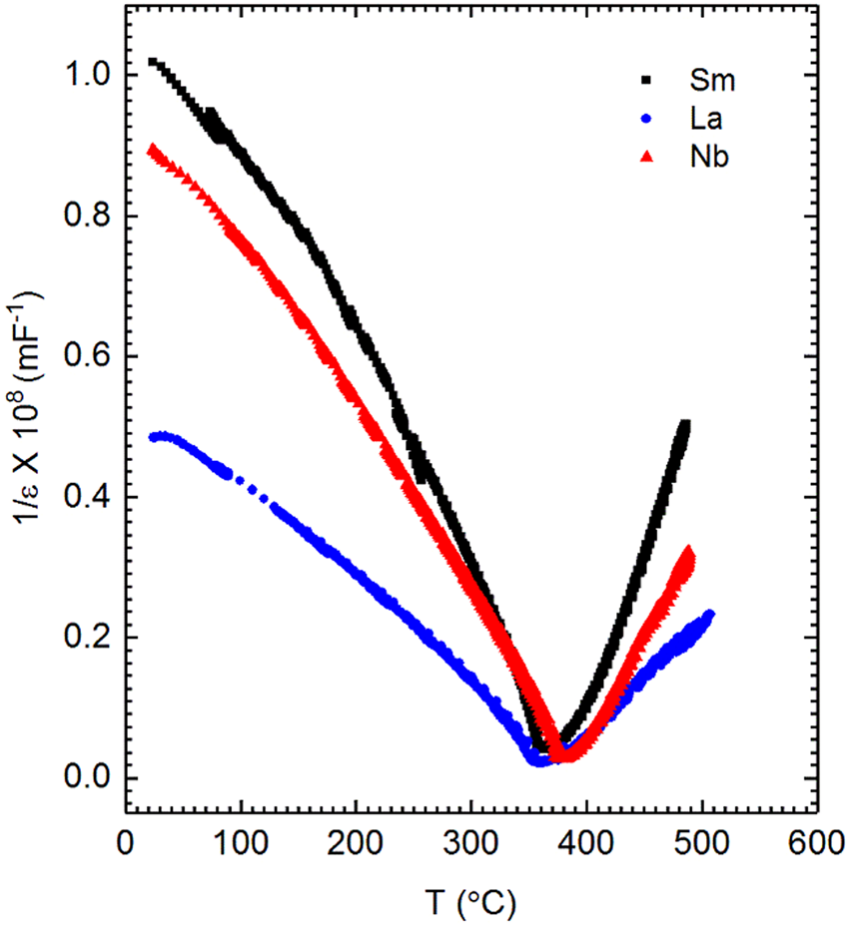
Reciprocal permittivity as a function of temperature for 2 at% Sm-, La- and Nb-doped PZT 50:50.

**Figure 9:**
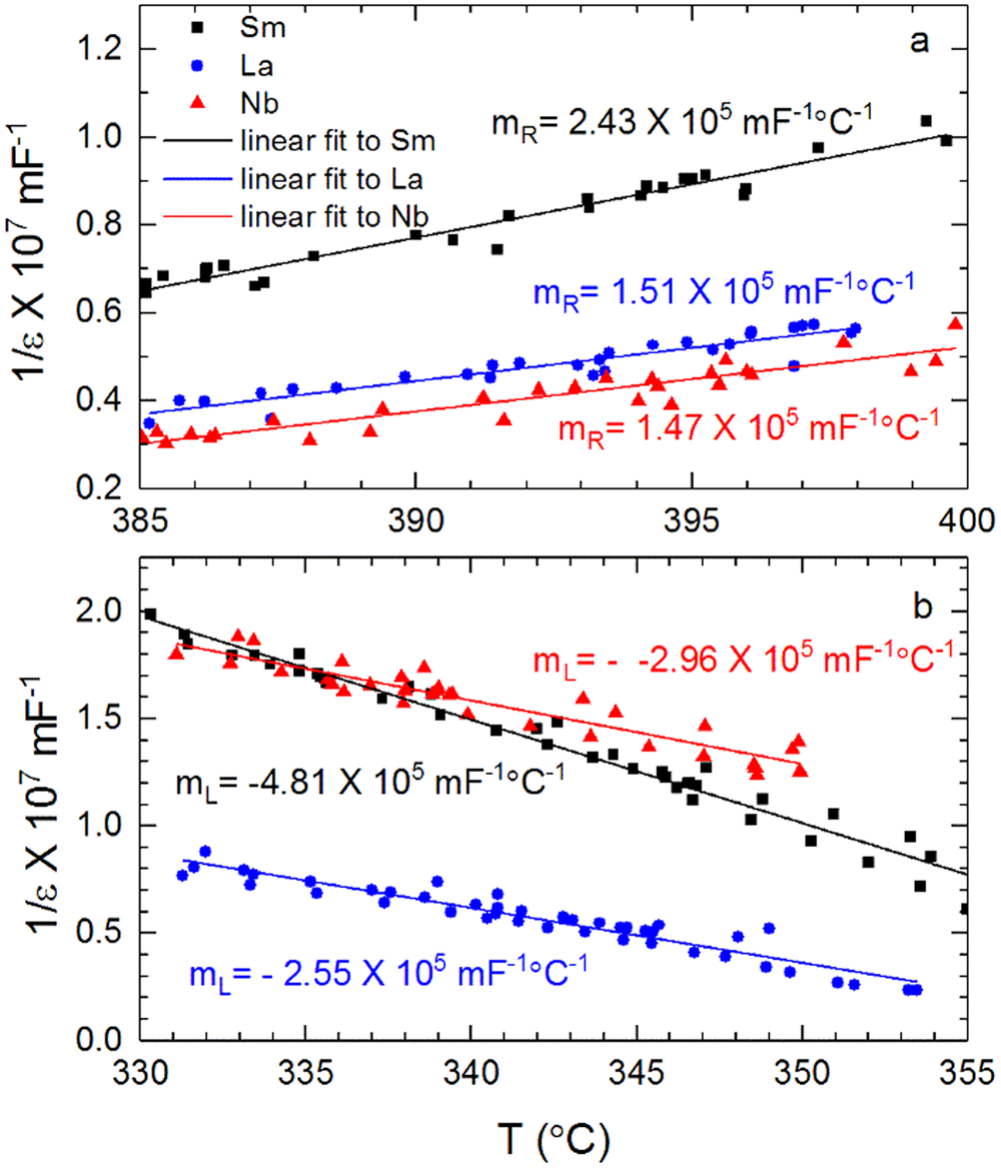
Linear fits to the (**a**) right and (**b**) left linear regions at the point of divergence of reciprocal permittivity plotted for 2 at% Sm-, La- and Nb-PZT 50:50.

In these equations, ε is permittivity, T is temperature, *T*_c_ is the Curie temperature and β, γ and δ are constants. The order of the phase transition can be determined from the variation of reciprocal permittivity as a function of temperature by calculation of the slopes of the reciprocal permittivity curve in the linear region at the point of divergence. For a first order phase transition, the absolute value of the ratio of the slopes of the left and right regions is 0.125. The variation of reciprocal permittivity as a function of temperature in this work is shown in Fig. 8, for Sm-, La- and Nb-doped PZT 50:50. The left and right linear regions at the point of divergence are plotted in Fig. 9. The ratio of the slopes is found to be approximately 0.5 for all the doped PZT 50:50 compositions as shown in Table 2. This value indicates that the phase transition is second order. Apparently, the simple continuum Landau-Devonshire theory and permittivity data alone are insufficient to describe the heterogeneous system examined herein.

**Table 2:** Ratio of the slopes of the linear fits to the left and right regions of reciprocal permittivity at the point of divergence.

Material	m_R_/-m_L_
2 at % Sm-doped PZT 50:50	0.5
2 at % La-doped PZT 50:50	0.5
2 at % Nb-doped PZT 50:50	0.6

## Discussion

Sm-doping results in unexpectedly large piezoelectric coefficients in the region of the *T*_c_. At temperatures above 250 °C, the *d*_33_ of Sm-doped PZT shows a rapid increase, approximately twice the values generated by the other donor dopants, La and Nb. The mechanisms for generating high piezoelectric coefficients in the present work are different from the mechanisms currently used to create commercial piezoelectric materials with high piezoelectric properties. Current commercial materials use a combination of donor doping (which is known to enhance domain wall motion at room temperature) and lowering of the *T*_c_ (which results in increased dielectric and electrostrictive properties at room temperature) to generate high piezoelectric coefficients. In the present work, three donor dopants were used, but only one yielded high piezoelectricity at elevated temperatures.

The high temperature piezoelectric behaviors of the materials used in this study and commercial PZT samples are also different, even though they are measured using the same methods and instruments. Some mechanisms for high piezoelectric coefficients in compositions near an MPB that have been previously offered include: (i) the existence of a lower symmetry monoclinic phase^15^ or the presence of monoclinic symmetry within microdomains^16^ and (ii) the presence of ferroelectric nanodomains^17^. Above ≈250 °C, the rate at which the *d*_33_ increases as a function of temperature is greater for all three PZT compositions used in this study relative to the commercial composition used by Anton *et al*.^15^. When the values of *d*_33_ begin to fall, the rate of the decay in the present work is also greater than that observed in the previously reported commercial PZT compositions. In the commercial PZT composition, the *d*_33_ decays from its maximum value to zero over a temperature range of ≈ 50 °C^18^. In the present work, the decay takes place over a temperature range of approximately 3 to 15 °C and resembles the decay shown by Bi_0.5_Na_0.5_TiO_3_^18^. This type of rapid decay in *d*_33_ during depoling has been associated with the existence of additional domain structures, such as low symmetry nanopolar domains in PZT^17^. However, in the present work, the doped PZT compositions closer to the MPB (PZT 50:50) and further from the MPB (PZT 45:55 and PZT 40:60) show similar decays in temperature-dependent *d*_33_. As Sm-doping gives rise to unexpectedly high *d*_33_ values in PZT materials at temperatures close to *T*_c_ across a range of Zr:Ti ratios from 50:50 to 40:60, MPB-related phenomena cannot fully account for the origin of the *d*_33_ values observed in the Sm-doped PZT at elevated temperatures.

We now consider other possible reasons for high piezoelectricity in Sm-doped PZT compared to the La- and Nb-doped samples in the Curie point region. The contributions to piezoelectric and dielectric properties of polycrystalline ferroelectrics can be divided into two categories: intrinsic and extrinsic. The intrinsic response of a ferroelectric is due to ionic and lattice displacements and thus originates from the piezoelectric and dielectric responses of individual domains. The extrinsic contributions to the material response are attributed to the presence of domain walls, defect dipoles and other effects not associated with a perfect crystal^19,20^. The total response of the material is determined by both the intrinsic and extrinsic responses. The intrinsic *d*_33_ and *ε*_*r*,33_ are related through the coefficient of electrostriction (Q_11_) and spontaneous polarization (*P*_s_) as per equation (3)^21^:

3 $${d}_{33}=2\,{\varepsilon }_{0}{\varepsilon }_{r,33}{{\rm{Q}}}_{11}{P}_{{\rm{s}}}$$

In the above equation, *ε*_0_ is the permittivity of vacuum. During the temperature-dependence studies, the *d*_33_ peak occurs at lower temperatures than the *ε*_*r*,33_ peak in all the PZT compositions (Fig. 1). Moreover, the maximum value of *ε*_*r*,33_ in the Sm-doped compositions is smaller than the maximum *ε*_*r*,33_ values of the La- and Nb-doped compositions for all Zr:Ti ratios. It is also important to note that there was no frequency dispersion observed in the real and imaginary permittivity. Therefore, discounting unexpected elastic effects, it may be inferred that the rapid increase in the temperature-dependent *d*_33_ of the Sm-doped composition above ≈250 °C is not due to its intrinsic permittivity, *ε*_*r*,33_.

**Figure 10:**
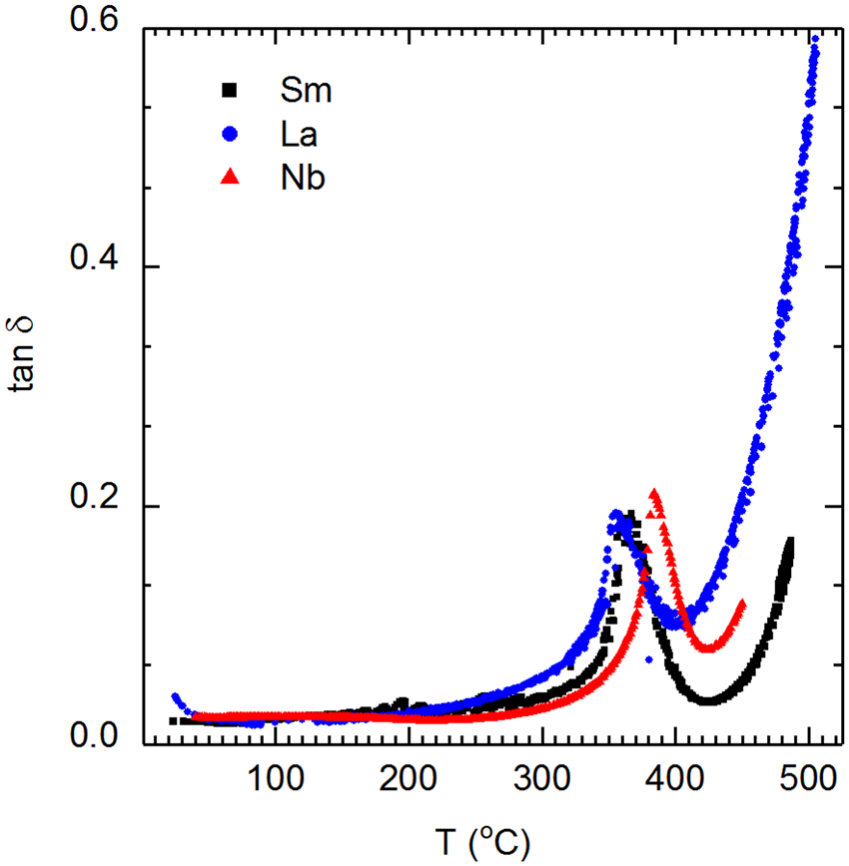
Variation of the dielectric loss tangent as a function of temperature for various PZT compositions.

The possibility that the valence of Sm changes from its nominal value of +3 to +2 at high temperatures is now considered. Sm^3+^ ion is a donor dopant, but in its +2 state, Sm^2+^ would be an isovalent dopant. A major effect of isovalent doping is enhancement of permittivity^22^. No atypical enhancement of permittivity is observed at temperatures above ≈250 °C (Fig. 1). Donor doping compensation for *p*-type conductivity is usually assumed for PZT materials^23^. As a consequence, if the valence of Sm changes from +3 to +2, it should result in both an increase in conductivity and dielectric loss tangent. Figure 10 shows the dielectric loss tangent as a function of temperature for Sm-, La- and Nb-doped PZT. An increase in loss tangent, which would be indicative of the presence of Sm^2+^, is not observed. Furthermore, Sm^2+^ is unstable^24^. Thus, it is unlikely that the valence of Sm changes from +3 to +2 at high temperatures. Substitution of Sm^3+^ on the B-site is also improbable because the six-coordinate ionic radius of Sm^3+^ (0.958 Å^25^) is 45% larger than that of the average B-site cation (0.66 Å).

Extrinsic effects are now considered as a possible explanation for the high *d*_33_ values observed in Sm-doped PZT in the region of *T*_c_. Tetragonal PZT has two types of domain walls: 180° and 90° walls. Extrinsic effects due to 90° domain wall motion are known to contribute to enhanced piezoelectric coefficients at room temperature^26^ and have been shown to contribute up to 70% of *d*_33_ in PZT^26–29^. It is known that donor dopants affect the defect structure in a way that enhances the mobility of domain walls, i.e. by changing the way in which the elastic and/or electric nature of the domain wall interacts with point defects and defect complexes. In the present work, three donor dopants resulted in similar room temperature piezoelectric properties, but markedly different behaviors at elevated temperature. Thus, the conventional ways of rationalizing the influence of donor doping on softening of piezoelectric properties is not directly applicable to the present results. It is also known that 90° domain wall reorientation increases exponentially with temperature^30^ and that it can be modeled by the Arrhenius equation. As a result, it is possible that 90° domain wall motion increases more rapidly in the Sm-doped PZT sample at temperatures above some critical energy threshold of ≈250 °C. The 180° domain walls can also contribute substantially to the *ε*_*r*,33_ of polycrystalline ferroelectrics, as recently shown by Fancher *et al*.^31^. However, as mentioned previously and shown in Fig. 1, the values of *ε*_*r*,33_ do not exhibit the same unexpected increase with temperature as the piezoelectric values. Therefore, 180° domain wall contributions are not anticipated to contribute to the high *d*_33_ values observed in Sm-doped PZT. Instead, the motion of 90° domain walls remains a likely contributor to the high piezoelectric properties.

The temperature-dependence of the crystal structure is a key to understanding the origin of the behavior. With increasing temperature, the *c/a* ratio of tetragonal PZT is known to decrease^6^, which may affect domain wall mobility and the extrinsic contribution to the piezoelectric coefficients. More specifically, the electric field-induced strain is a function of both *c/a* and extent of 90° domain wall motion, as shown by Tutuncu *et al*.^32^. In ref.^32^, as the *c/a* ratio decreased, the extent of 90° domain wall motion increased disproportionately, leading to a net enhancement in electric-field-induced strain coefficients. In the present work, the *c/a* of the tetragonal phase decreases with increasing temperature, which may contribute, at least in part, to the increasing *d*_33_ coefficients measured at elevated temperatures. However, the thermal cycling experiments demonstrate that the enhancement decreases after cycling (Fig. 2), suggesting that *c/a* ratio changes cannot completely explain the high-temperature *d*_33_ measurements in Sm-doped PZT.

Given that heating causes depoling, one must also recognize that these experiments may be rate dependent, as depoling may occur progressively during the heating process. In fact, a competition may exist between thermal depoling (dominating at slower heating rates), increased mobility of domain walls (dominating at faster heating rates due to thermal activation) and density of domain walls. The defects created by Sm-doping could also influence this behavior, because defects are known to migrate to or stabilize domain walls. These competing effects make it a complex system in which extrinsic and intrinsic effects interplay.

In systems in which multiple phases coexist^33^, high piezoelectric coefficients have been associated with mechanisms such as interphase boundary motion^34^ (the motion of the interface between the phases) and the concept that materials containing multiple polymorphs can better accommodate strain by allowing for more overall deformation strain in polycrystalline aggregates^35,36^. Though interphase boundary motion has been shown to be frequency dependent^37^, it may still be of some significance in the kHz frequency range at which the piezoelectric coefficients were measured in this study.

The motion of 90° domain walls is also different in single-phase vs. two-phase materials. Significant 90° domain wall activity has been directly measured using X-ray diffraction during application of electric fields in mixed-phase Bi(Me)O_3_-PbTiO_3_ compositions (where Me represents a metal cation)^37,38^. These materials have a secondary polymorph that can be characterized as pseudo-cubic. We first note the correlation of enhanced piezoelectric properties and two-phase coexistence in these prior studies^37,38^, demonstrated at room temperature. In the present work, the correlation between extraordinarily high d_33_ and two-phase coexistence is shown at elevated temperatures and the enhanced response may benefit from the thermal energy^30,39^. In the Sm-doped PZT compositions at elevated temperatures, evidence indicates the motion of 90° domain walls and their contribution to piezoelectric properties due to the coexisting pseudo-cubic phase, which may be associated with better strain accommodation or interphase boundary motion.

Materials at phase transitions and near boundaries of polar and nonpolar phases often show high intrinsic electromechanical and dielectric responses; the coexistence of a tetragonal and pseudo-cubic phase in the present work may indicate proximity to such behaviors^40^. However, the permittivity does not show an enhanced response at these temperatures because non-180° domain walls do not contribute as substantially as 180° domain walls to the *ε*_*r*,33_ of polycrystalline ferroelectrics^31^. Alternatively, it is likely that the coexistence of polymorphs in the Sm-doped PZT compositions enable these materials to have a larger extrinsic (e.g., domain wall) contribution to the properties due to better strain accommodation during deformation and/or interphase boundary motion. As La- and Nb-doping do not result in a mixed phase material at high temperatures, the extent of 90° domain wall motion and the resulting strain should be comparably restricted. Consequently, temperature dependent *d*_33_ in La- and Nb-doped PZT would not increase as rapidly as observed in Sm-doped PZT (Fig. 1).

The crystallochemical reasons for the unique phase coexistence and phase transition behavior of Sm-PZT (relative to Nb and La-doped PZT) are now considered. PZT has a perovskite crystal structure typified by the formula ABO_3_ (shown in Supplementary Information Fig. S3). In PZT, the ionic radius of the 12-fold coordinated host A-site ion, Pb^2+^, is 1.49 Å. The 12-fold coordinated ionic radius of A-site dopants La^3+^ and Sm^3+^ are 1.36 Å^25^ (6% smaller than host ion Pb^2+^) and 1.24 Å^25^ (14% smaller than host ion Pb^2+^), respectively. The average ionic radius of the host B-site atoms Ti^4+^ (ionic radius 0.605 Å^25^) and Zr^4+^ (ionic radius 0.72 Å^25^) is 0.66 Å. The ionic radius of Nb^5+^ is 0.64 Å^25^ (3% smaller than the average ionic radius of the host Ti^4+^ and Zr^4+^ ions). When a dopant atom replaces a host atom of a different size, it induces strain. A framework in which to describe this strain simplistically is the concept of internal strain or “chemical pressure.” Though the bonding type, bonding directionality and elastic compliance of the lattice may affect how this chemical pressure is realized at longer length scales, the concept can generally describe the effects of differing ionic radii. As the ionic radius of Sm^3+^ deviates most from the host values, the Sm-doped PZT compositions can be considered to have the most chemical pressure. Ahart *et al*.^35^ suggest that the chemical pressure created due to doping varies monotonically with the pressure needed for phase transformation. For example, Zeches *et al*.^41^ report the observation of strain-induced phase transformation in thin film BiFeO_3_ perovskite, in which there is a hydrostatic strain component. Chemical pressure driven phase transformations have been shown through first principles calculations in other materials systems^42^. The combination of the chemical pressure resulting from Sm-doping and increased temperature may influence the tetragonal ferroelectric to cubic paraelectric phase transition, in this case allowing for the formation of a mixed tetragonal and pseudo-cubic phase over a defined temperature range.

The results of this work suggest that the coexistence of tetragonal and pseudo-cubic polymorphs in Sm-doped PZT are the requisite condition for the high piezoelectric coefficients at elevated temperatures. This coexistence then facilitates 90° domain wall motion in the tetragonal phase, an interpretation consistent with data in refs^37,38^ and possibly interphase boundary motion. The thermal cycling experiments demonstrate that the nature of the contribution can change, likely due to changes in phase fractions, domain wall density, or defects with cycling. The concept of chemical pressure in Sm-doped PZT is used to consider the origin of the two-phase coexistence, a feature that could be replicated in other ferroelectric compositions.

In summary, the behavior of *d*_33_ as a function of temperature was measured for Sm-, La- and Nb-doped PZT across a range of Zr:Ti ratios in order to understand the impact of chemical substitution on the high temperature piezoelectric coefficients of donor-doped PZT. It was found that Sm-doping results in high temperature piezoelectric coefficients, which are much greater than those obtained by doping with La^3+^ or Nb^5+^ across all the Zr:Ti ratios studied. Enhanced permittivity and MPB-related phenomena cannot account for the high piezoelectricity in the Sm-doped PZT compositions. It is suggested that Sm-doping gives rise to the greatest chemical pressure in PZT, relative to La^3+^ and Nb^5+^, as the ionic radius of Sm deviates most from the ideal value for a cubic perovskite. This large internal stress caused by Sm^3+^ may change the ferroelectric to paraelectric phase transitions characteristics in Sm-doped PZT allowing for a mixed tetragonal and pseudo-cubic phase to form at temperatures below *T*_c_. The coexistence of a pseudo-cubic phase with a ferroelectric tetragonal phase may allow higher strain accommodation capacities in Sm-doped PZT compared to the La- and Nb-doped counterparts and therefore have a greater potential for interphase boundary motion. With increasing temperature, the energy available for 90° domain wall motion increases exponentially for all the PZT compositions. However, the Sm-doped PZT compositions have a larger capacity to accommodate strain from an increase in 90° domain wall mobility and the possibility of interphase boundary motion. Hence, the Sm-doped compositions have high temperature piezoelectric coefficients that are roughly twice those obtained by doping with La and Nb. Thermal cycling studies further reinforce that the origin of the high *d*_33_ in Sm-doped PZT is extrinsic in nature.

## Methods

### Sample synthesis

Disc shaped pellets (10 mm diameter and 1.5 mm thick) were produced using conventional solid state synthesis techniques. Silver paste was painted onto to the flat surfaces as electrodes and the samples were then fired at 550 °C for 30 min. The samples were electrically poled to saturation at room temperature by applying an electric field just below the breakdown field of the material. The poled disc samples were used for both *d*_33_ and *ε*_*r*,33_ measurements. All samples were aged for at least one week at room temperature prior to the measurements in order to mitigate the potential effects of initial aging on ferroelectric behavior^43^. The high-field Polarization-Electric Field (P-E) and Strain-Electric Field (S-E) loops for Sm-, La- and Nb-doped PZT 50:50 are shown in Supplementary Information Fig. S4(a) and (b), respectively.

### Electrical measurements

The temperature dependent small signal *d*_33_ was measured on disc shaped samples using a custom-built apparatus based on Doppler vibrometry^10,44^. A sinusoidal voltage of ±10 V at a frequency of 1 kHz was used as an input signal. The *ε*_*r*,33_ was measured by an LCR meter using an oscillation voltage of 1 V and frequency 1 kHz. Poled samples were placed into a furnace with two thermocouples to cross-check the uniformity of temperature around the sample. Displacements induced in the sample due to the converse piezoelectric effect were determined by the Doppler effect with a precision of ±1 pm, though the actual resolution is limited by background noise at >10 pm. Full details are published in Leist *et al*.^10^. The sample was placed in a furnace and heated at a ramp rate of ≈2 °C/min during both the *d*_33_ and *ε*_*r*,33_ measurements^45^.

### XRD measurements and analysis

For the high resolution XRD measurements, the sintered disc shaped pellets were crushed into a powder. The powder was annealed at 600 °C for 3 h in a closed alumina crucible to alleviate residual intragranular stresses. High resolution XRD patterns were measured on beamline 11-BM of the Advanced Photon Source at Argonne National Laboratory. A monochromatic X-ray beam with a wavelength of 0.41295(3) Å and a 2θ range of 0–20° with a 2θ step size of 0.0001° was used. The sample powders were packed into a 0.3 mm diameter quartz capillary and spun at 60 Hz about the axis of the capillary to increase powder averaging. The samples were heated from room temperature to 500 °C at a ramp rate of 2 °C/min. XRD patterns were measured for 30 min at six fixed temperatures (with uncertainties of ±1 °C) during cooling in the following order: 500 °C, 360 °C, 332 °C, 308 °C, 290 °C and 250 °C. The peak fitting procedure for segments of the diffraction patterns was carried out using LIPRAS^46^, a peak fitting GUI that utilizes the Curve Fitting Toolbox^TM^ within MATLAB (MathWorks, R2016b).

The Rietveld refinements of the entire diffraction patterns were carried out using GSAS^47^. In the refinements (shown graphically in Figs 6 and 7), 12 background parameters in the shifted Chebyshev model were used. A combination of profile functions 3 and 4 were utilized. Profile function 4 includes the Stephens model for asymmetry^48^ which was used to model the asymmetric peak shapes, particularly in the refinements using the tetragonal space group. The parameters refined were the 2θ zero, scale factor, lattice parameters, atomic positions in the tetragonal phase and isotropic displacement parameters. For all refinements, nominal stoichiometries of the compositions were assumed. During refinement, Zr and Ti were constrained to the same atomic position and displacement parameter in order to satisfy structure factor requirements in Rietveld refinement. For the same reason, Sm and Pb were both constrained to the (0, 0, 0) atomic position and the same isotropic displacement parameter.

### Data availability

The data supporting the main findings of this study are available from the corresponding author on request.

## Electronic supplementary material

Supplementary Information
