# Supplementary material for: Unexpectedly high piezoelectricity of Sm-doped lead zirconate titanate in the Curie point region
Source: Sci Rep. 2018 Mar 7;8:4120. doi: 10.1038/s41598-018-22566-5 (PMC5841290; doi:10.1038/s41598-018-22566-5)

Supplemental Information for

Unexpectedly high piezoelectricity of Sm-doped lead zirconate titanate in the Curie point region

Shruti B. Seshadri[1,2#], Michelle M. Nolan[1,3], Goknur Tutuncu[2], Jennifer S. Forrester[4], Eva Sapper[5], Giovanni Esteves[2], Torsten Granzow[6], Pam A. Thomas[7], Juan C. Nino[1], Tadej Rojac[8], and Jacob L. Jones[2*]

[1]Department of Materials Science and Engineering, University of Florida, Gainesville, Florida, 32611, USA.

[2]Department of Materials Science and Engineering, North Carolina State University, Raleigh, North Carolina 27695, USA.

[3]Department of Chemistry, University of Florida, Gainesville, Florida, 32611, USA.

[4]School of Chemical and Process Engineering, University of Leeds, Leeds, LS2 9JT, United Kingdom.

[5]Institute of Materials Science, Technische Universität Darmstadt, 64287 Darmstadt, Germany.

[6]Materials Research & Technology Department, Luxembourg Institute of Science & Technology, L-4362 Esch/Alzette, Luxembourg.

[7]Department of Physics, University of Warwick, Coventry CV4 7AL, United Kingdom.

[8]Jozef Stefan Institute, Jamova cesta 39, 1000 Ljubljana, Slovenia.

[#]Currently at Intel Corporation, Oregon, USA.

Supplementary Information

Supplementary Table S1. Rietveld results of $Pb_{0.97}Sm_{0.02}Zr_{0.5}Ti_{0.5}O_3$ showing the refined atomic positions at all temperatures (with e.s.d.s). Extended Criteria of Fit indices are also supplied.

Temperature (°C)	Atomic position B (z)	Atomic position O1 (z)	Atomic position O2 (z)	Criteria of fit
250	0.5337(6)	0.419(1)	-0.042(5)	R_p = 9.00%<br>R_{wp} = 11.20%<br>Chi^2 =2.804
290	0.5280(6)	0.427(1)	0.034(5)	R_p = 7.90%<br>R_{wp} = 9.55%<br>Chi^2 = 2.071
308 (T only)	0.5250(7)	0.438(2)	0.041(4)	R_p = 8.43%<br>R_{wp} = 10.25%<br>Chi^2 = 2.388
308 (T from T+C model)	0.5280(5)	0.446(2)	0.049(2)	R_p = 5.40%<br>R_{wp} = 6.65%<br>Chi^2 = 1.005
332 (T only)	0.483(1)	0.433(2)	-0.016(7)	R_p = 6.82%<br>R_{wp} = 8.08%<br>Chi^2 = 1.519
332 (T from T+C model)	0.470(1)	0.575(2)	-0.041(3)	R_p = 5.70%<br>R_{wp} = 7.11%<br>Chi^2 = 1.176
360	-	-	-	Rp = 8.64%<br>Rwp = 10.54%<br>Chi2 = 2.648
500	-	-	-	Rp = 7.59%<br>Rwp = 8.8%<br>Chi2 = 1.1901

Supplementary Table S2. Rietveld results of $Pb_{0.97}Sm_{0.02}Zr_{0.5}Ti_{0.5}O_3$ showing the refined isotropic displacement parameters at all temperatures: **tetragonal phase**. All values are multiplied by 100 (with e.s.d.s)

Temperature (°C)	A-site U_{iso}	B-site U_{iso}	O1-site U_{iso}	O2-site U_{iso}
250 (T)	4.21(2)	1.34(3)	2.6(2)	6.8(4)
290 (T)	4.70(2)	1.49(3)	2.3(1)	6.7(3)
308 (T only)	4.81(2)	1.39(3)	2.7(2)	4.1(3)
308 (T from T+C model)	4.43(3)	0.44(3)	2.6(2)	0.7(2)
332 (T only)	5.05(1)	1.44(3)	1.1(2)	6.5(5)
332 (T from T+C model)	4.14(4)	0.63(6)	2.9(3)	0.9(4)

Supplementary Table S3. Rietveld results of $Pb_{0.97}Sm_{0.02}Zr_{0.5}Ti_{0.5}O_3$ showing the refined isotropic displacement parameters at all temperatures: **pseudocubic phase**. All values are multiplied by 100 (with e.s.d.s)

Temperature (°C)	A-site U_{iso}	B-site U_{iso}	O1-site U_{iso}
308 (C from T+C model)	5.17(6)	4.8(1)	4.9(5)
332 (C from T+C model)	5.99(6)	2.19(9)	4.7(2)
332 (C only)	4.74(1)	1.69(2)	5.32(8)
360	5.47(2)	1.60(2)	4.00(8)
500	5.88(1)	1.68(2)	3.87(6)

Supplementary Figure S1: Variation of Fig. 5 in main manuscript that calculates R_p and R_{wp} within the shaded grey region for data plotted that is individually normalized by its maximum intensity. This illustrates the observed data as obs/max(obs) and the calculated data as calc/max(calc). This method of plotting normalized data by respective maximum observed intensity highlights that using 3 symmetric peaks is the better model for the measured data. The purpose of normalizing the obs and calc separately is to highlight the discrepancy in both data sets. If the calc matches the obs then the following relationship should hold:

$$\frac{Obs}{\max(Obs)} = \frac{Calc}{\max(Calc)}$$

The bottom right plot shows the squared error within the shaded grey region to further demonstrate that 3 symmetric peaks are needed to have the best fit to the measured data.

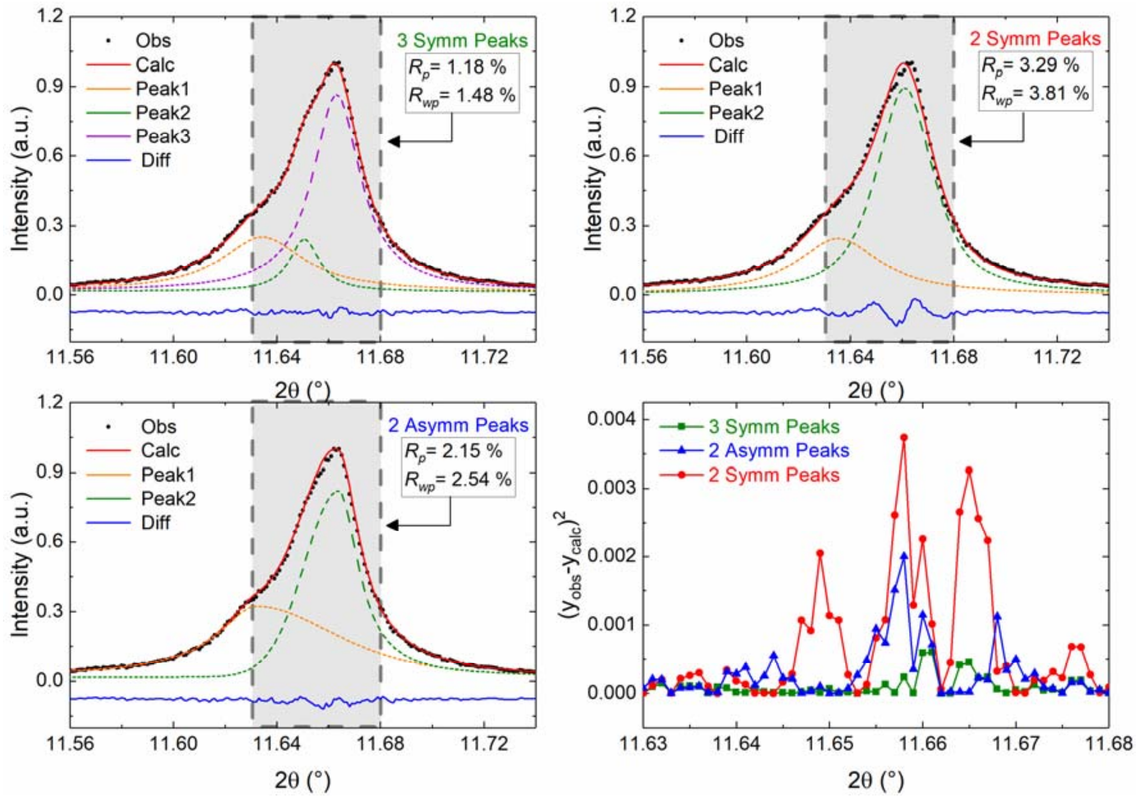

Supplementary Figure S2: Asymmetric Strain profile

This figure shows that the distinct shoulder on the left side of the 200 tetragonal reflection is due to an additional peak and not a result of domain wall scattering. This figure was created by peak fitting of the left shoulder of the 002 reflection and mirroring the result to create a symmetric 002 reflection. The same was done for the 200 reflection in which the right-hand side of the peak was fit then mirrored over to the left to produce a symmetric reflection. The subtraction of both peaks to the overall data represents the blue difference curves, which points towards the indication that another peak is needed to account for the intensity at the center that is present due to phase coexistence.

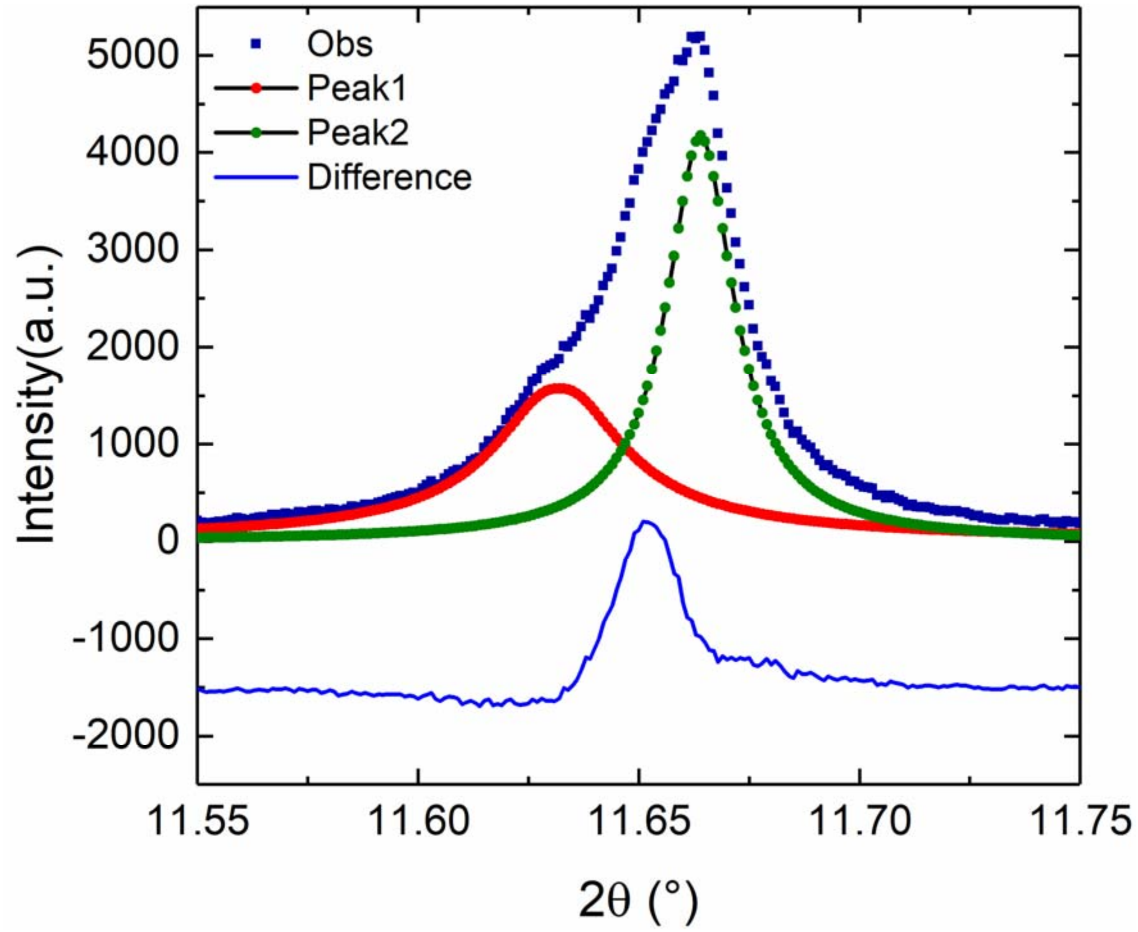

Supplementary Figure S3: Perovskite crystal structure representing tetragonal PZT (ABO_3)

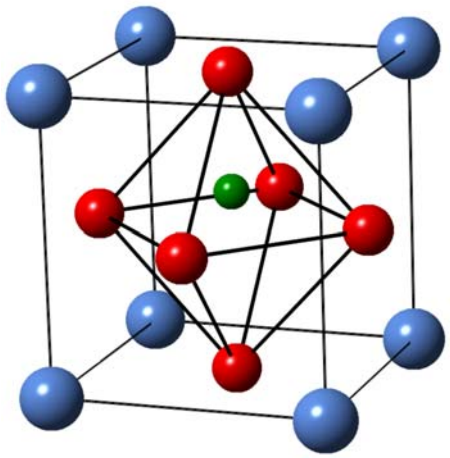

Blue sphere = A atoms = Pb 97% / Sm 2%

0, 0, 0

Green sphere = B atoms = Zr 50% / Ti 50%

0.5, 0.5 0.5 ±z (in pseudocubic phase z = 0)

Red sphere = O atoms = Oxygen 100 %

O1: 0.5, 0.0, 0.5 ±z

O2: 0.5, 0.5, 0 ±z (in pseudocubic phase z = 0)

Supplementary Figure S4: (a) Polarization, and (b) strain in response of Sm-, La-, and Nb-doped PZT 50:50 to an applied bipolar triangular waveform electric field of amplitude 3.5 kV/mm and frequency 1 Hz. From Figure S2(a), the coercive fields are Sm = 2.5 kV/mm, Nb = 2.3 kV/mm, and Nb = 3.9 kV/mm.

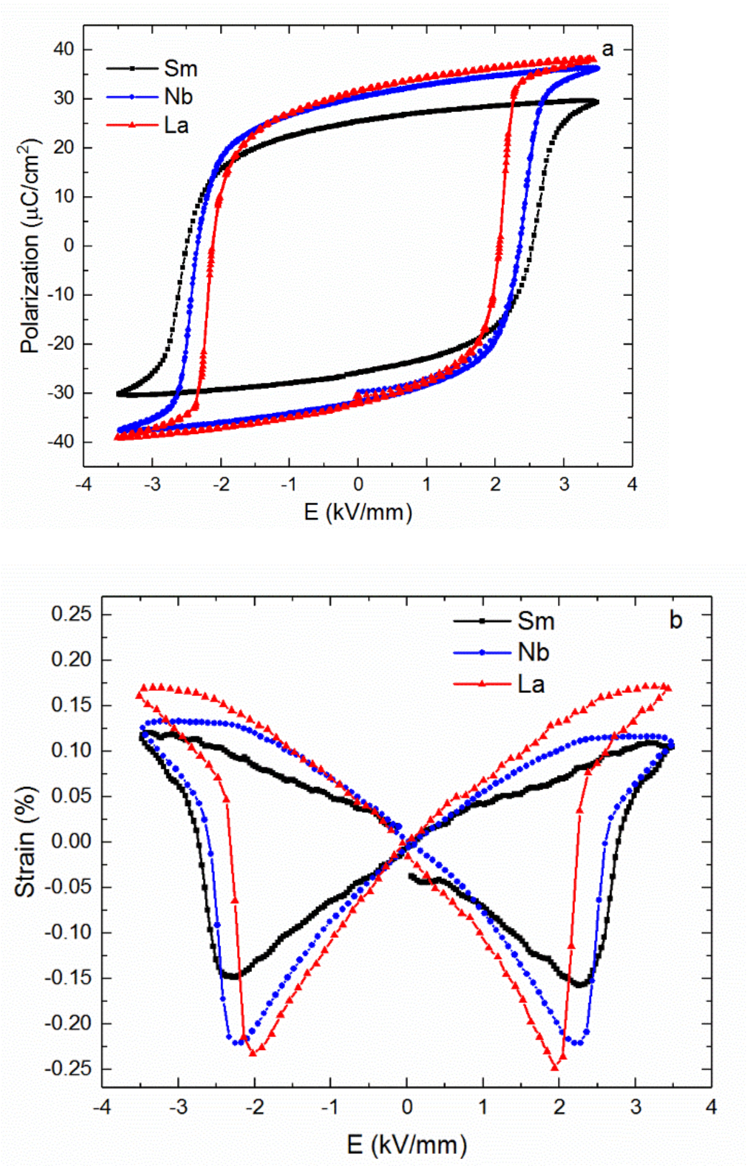

Supplement: Supplementary file 1 — Supplementary Information [file 41598_2018_22566_MOESM1_ESM.pdf]
